# Severe Trichotillomania: An Unusual Trigger of Recurrent Diabetic Ketoacidosis

**DOI:** 10.7759/cureus.21384

**Published:** 2022-01-18

**Authors:** Larissa Check, Gabriela Figueroa, Lidice Galindo, Jamie Pham, Robert Sherertz

**Affiliations:** 1 Internal Medicine, Grand Strand Medical Center, Myrtle Beach, USA; 2 Internal Medicine/Infectious Disease, Grand Strand Medical Center, Myrtle Beach, USA

**Keywords:** obsessive-compulsive symptoms, atypical rash, atypical, severe diabetic ketoacidosis, diabetic ketoacidosis, trichotillomania

## Abstract

Diabetic ketoacidosis (DKA) is a life-threatening complication of diabetes that is most often seen in patients with type 1 diabetes mellitus. Current DKA management focuses on rapid treatment to prevent acute complications, educational intervention, and early discharge. However, patients with mental health conditions face additional barriers to establishing control over their diabetes and may be hospitalized often for DKA recurrence. Understanding a patient’s mental health and intervening where necessary may be a crucial step in the effective treatment of DKA. We present a case of recurrent DKA in a young male who suffered from severe trichotillomania. Trichotillomania is a mental health disorder in which an individual has the compulsion to pull on hair because it feels good. By doing so, the skin barrier is compromised. This can lead to disfiguring lesions that can be very distressing for the individual; however, they report an inability to control the compulsion to stop pulling their hair. In our case, the patient disrupted the skin barrier, leading to increased susceptibility for recurrent infection and DKA.

## Introduction

Diabetic ketoacidosis (DKA) is a potentially life-threatening emergency that occurs as a complication of uncontrolled diabetes. While this is often seen in those with type 1 diabetes, it can also manifest in those with type 2 as well due to insufficient insulin levels [1]. This prevents the body from performing normal metabolic functions, as glucose is unable to enter the cells and remains in the bloodstream. The body interprets the lack of intracellular glucose as being in a state of starvation and will respond by breaking down stored glycogen and fats to produce usable glucose. This breakdown of fats produces ketone acid byproducts that cause metabolic acidosis [2]. Often, the body will counteract this metabolic acidosis with respiratory alkalosis, the action of hyperventilation with rapid shallow breathing, also known as Kussmaul respirations [2]. In addition, to counter hyperglycemia, the kidneys will excrete the excess glucose through osmotic diuresis along with water and electrolytes [1]. This osmotic diuresis causes hypovolemia and electrolyte imbalances. Acute kidney injury can occur if the hypovolemia is severe enough. Furthermore, due to dehydration, the blood will become more concentrated, which causes hyperosmolarity. Hyperosmolarity in conjunction with electrolyte imbalance can ultimately cause cardiac arrhythmias and coma if severe enough or left untreated long enough.

The most common causes for DKA are new-onset diabetes, improper insulin administration, or some kind of body stress such as infection/sickness or trauma [1-2]. However, DKA can also be caused by other factors like medications, severe mental illnesses, drug or alcohol abuse, and starvation. Oftentimes, these patients will present with symptoms such as polyuria, polydipsia, fruity breath, abdominal pain, nausea, vomiting, weakness, drowsiness, tachycardia, and hypotension. Regardless of the cause, all patients must be given insulin, fluid, and electrolyte restoration. If there is an underlying cause, it must be addressed immediately in order to improve survival and decrease recurrent hospitalization. In this case, we report trichotillomania as a possible cause of recurrent DKA.

## Case presentation

We present a case of a 26-year-old male with a past medical history of uncontrolled type 1 insulin-dependent diabetes and surgical history of several abdominal surgeries after a car accident 12 years ago who presented with nausea, vomiting, and diarrhea for the past two days. The patient stated that he usually has a loose stool once every three days but this had increased in frequency prior to admission. He expressed concerns of non-bloody emesis and noted it was correlated with blood sugars above 300s. When his symptoms worsened, he decided to be evaluated in the emergency department. On arrival, the patient’s blood glucose by finger stick was 736 and he reported that this was his second DKA episode in the past month. He had several lesions on bilateral anterior shins, the right knee, right gluteal, and crown of the scalp. When asked about these findings, the patient offered no explanation as to how the lesions came to be but reported that they have been present for several months. The lesions were not painful but were itchy. He denied any symptoms of arthritis, vision changes, headache, lightheadedness, chest pain, shortness of breath, fever, or chills. There was no pertinent family history of inflammatory bowel disease, arthritis, or autoimmune conditions.

Vitals revealed tachycardia and low blood pressure 91/52. Initial workup with chemistry revealed sodium 151 (135-146 mmol/L), potassium 4.9 (3.5-5.1 mmol), carbon dioxide 12 (22-32 mmol/L), anion gap 32 (3.0-11.0 mEq/L), creatinine 1.30 (0.7-1.5 mg/dL), estimated glomerular filtration rate (GFR) >60 (modification of diet in renal disease equation, MDRD) (>=60), glycated hemoglobin HbA1c (12.9; 3.8-5.6%), and lactic acid 1.8 (0.7-2.1 mmol/L). Hematology revealed WBC 15.8 (3.7-10.1 K/mm^3^), Hgb 6.4 (14.0-16.4 gm/dl), hematocrit (Hct) 24.7 (40.0-47.2%), mean corpuscular volume (MCV) 76.3 (81.8-94.6 fL), platelet count 655 (150-400 K/mm^3^). Serum acetone and urine ketones were positive. Follow-up studies revealed iron 14 (37-181 mcg/dL), iron saturation 4 (15-50%), ferritin 9.3 (26.0-388.0 ng/mL), c-reactive protein 14.40 (0-0.99 mg/dL), and erythrocyte sedimentation rate (ESR) >140 (0-15 mm/hour). A peripheral smear showed slight polychromasia, anisocytosis but no schistocytes. The patient was typed and screened in due to severe anemia. Imaging studies, such as chest radiograph and CT abdomen, were negative for underlying infectious etiology such as pneumonia, intra-abdominal abscess, or colitis.

On the initial physical exam, the patient appeared well-nourished, non-toxic, and in no apparent acute distress. The abdomen was soft with mild tenderness to palpation over the left lower quadrant. There was no palpable organomegaly or visible ascites with normal bowel sounds to auscultation. Skin findings were remarkable for a single, well-demarcated, 5 cm, circular ulcer with exposure to the crown of the scalp, without purulence or surrounding erythema, edema, or drainage. Multiple oblong ulcerative lesions were noted on the bilateral lower extremities on the lateral sides, knee, and right gluteal area. Gross sensation and range of motion in the upper and lower extremities were unremarkable. Muscle strength of bilateral upper/lower extremities was 5/5, equal, and symmetrical. The patient denied homicidal or suicidal ideation and was perceived to have normal affect, insight, and judgment.

Management for DKA was initiated immediately with resuscitation measures for optimal stabilization. The patient was started on the hospital DKA protocol of 0.1 U/kg/hour of intravenous (IV) insulin, normal saline at 500 cc/hr, and 30 mEq of potassium repletion. Blood cultures, urine cultures, and wound cultures were collected on admission. Broad-spectrum antibiotics were initiated as the patient met systemic inflammatory response syndrome (SIRS) criteria and sepsis could not be ruled out. Furthermore, he received a unit of packed red blood cells due to acute iron deficiency anemia.

**Figure 1:**
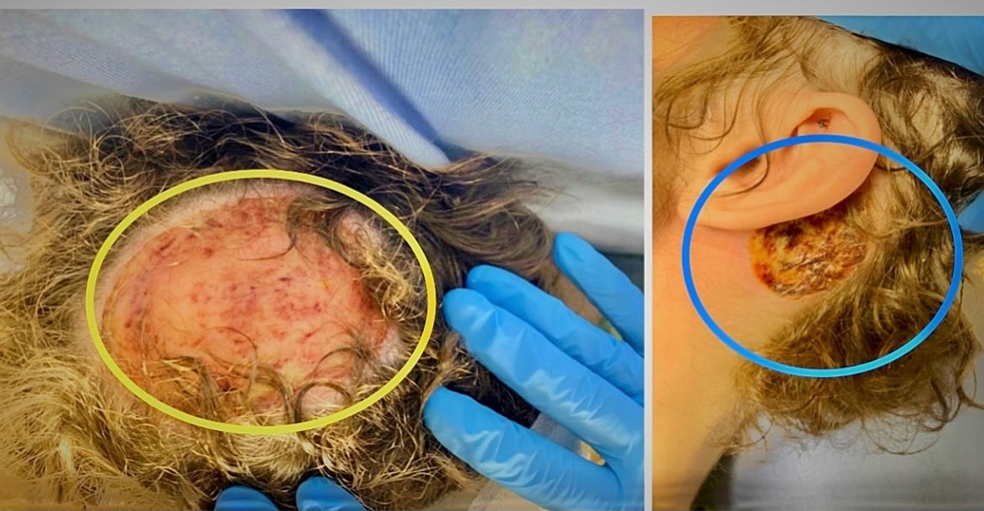
Erosion of the entire crown of the scalp to expose the skull (yellow circle). Another lesion was noted behind the right ear (blue circle).

**Figure 2:**
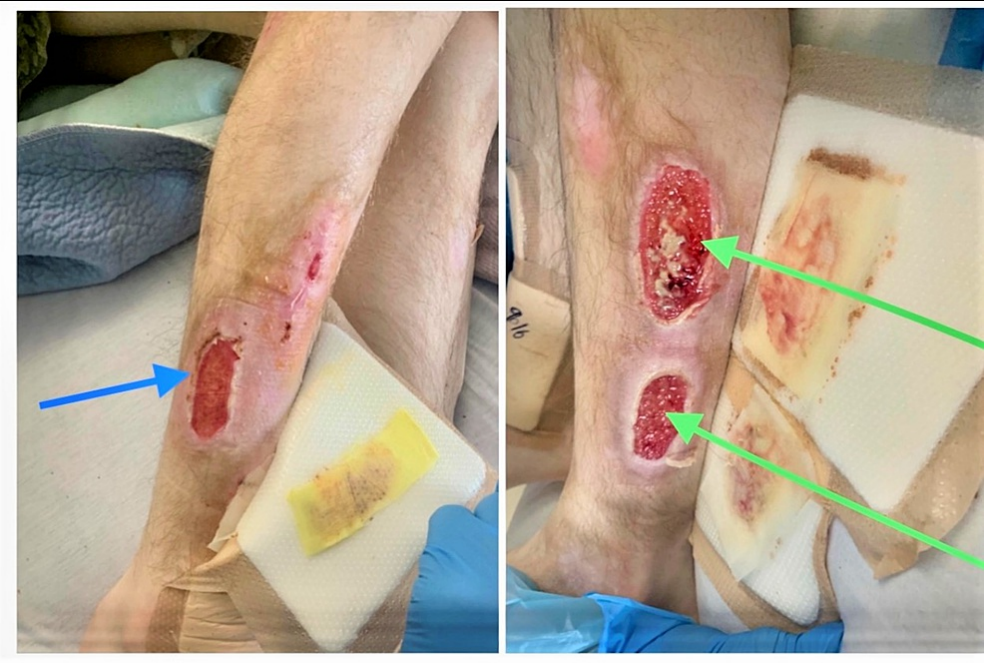
A large oval-shaped ulcerative lesion was noted on the lateral right leg (blue arrow) and two more lesions on the lateral left leg (blue arrows).

The initial differential diagnosis of a young type 1 diabetic with recurrent DKA, microcytic anemia, persistent granulomatous non-healing lesions, and recurrent diarrhea was broad and concerning for pyoderma gangrenosum in addition to infectious causes of ulcerative lesions, malignancy, and various forms of vasculitis. While reviewing the photographs, the very smooth borders of the lesions in addition to the lesions being at various stages of healing raised suspicion of the possibility of self-injury and repetitive behavior. Images of the skin lesions are shown in Figures 1-2.

Upon further interviewing the patient did admit to “picking on his skin” because it relieved anxiety and was a means of coping with past trauma. Furthermore, he stated the lesions were not painful and the act of picking on the skin and hair actually felt good and relaxing. He had never sought psychiatric help for this and was scheduled to start visiting a clinical psychologist at the behest of his father. He specifically denied the intention to cause self-harm, however, his unhealthy coping mechanism did result in self-injury. Although the patient reported self-injury, gastroenterology was consulted for the acute anemia, diarrhea, and possibility of pyoderma gangrenosum as a differential. Although it is probable he could have an underlying pyoderma, it was most likely the acute blood loss was the result of chronic trichotillomania. He was also seen by a psychiatrist and prescribed a mood stabilizer as he reportedly tried and failed SSRIs in the past. He was scheduled for follow-up with cognitive-behavioral therapy (CBT), which included virtual habit reversal training (HRT) in addition to group therapy.

## Discussion

Trichotillomania falls under the category of obsessive-compulsive disorders characterized by the recurrent pulling of hair and resulting in functional impairment [3]. Prevalence is between 1% and 3%; onset is usually during childhood or adolescence with female predominance [3-4]. There is high comorbidity with anxiety and mood disorders. While the exact pathophysiology is still being investigated, there is an association with SAPAP3 protein, including deletion of this gene associated with increased self-grooming behavior in animal models [4]. There is also a reported association with the SLITRK1 gene [5]. The hair pulling leads to alopecic plaques, with strands of hair of various lengths and uninflamed skin. According to the Diagnostic and Statistical Manual of Mental Disorders, 5th Edition (DSM-V), trichotillomania is categorized as an impulse control disorder and diagnostic criteria include hair loss as a result of recurrent hair pulling, repeated attempts to stop or decrease hair-pulling behavior, hair-pulling that causes significant clinical distress, hair-pulling not explained by any other medical condition, and hair-pulling not explained by another mental disorder [3]. Biopsy with histopathology is certainly warranted in cases where the etiology is unclear or there is underlying suspicion for other medical causes such as pyoderma gangrenosum. A biopsy is favored especially when dermatological conditions cannot be ruled out and this may include non-scarring and scarring alopecia.

Trichotillomania diagnosis is made clinically especially when the patient reports repetitive hair-pulling behavior that cannot be explained by any medical reason and causes marked functional impairment. Drug-induced trichotillomania has been documented in cocaine abusers and patients with attention deficit hyperactivity disorder (ADHD) on stimulants. It is reported as a rare neuropsychiatric side effect of Adderall, a psychostimulant medication for the treatment of ADHD [6]. Similarly, cocaine use can lead to hair-pulling behavior 30 minutes after smoking and is not associated with tactile hallucinations or underlying skin disorder [7]. Both examples of drug-induced trichotillomania reported the disappearance of symptoms with discontinuation of the drug and no evidence of recurrence.

First-line treatment is cognitive behavior therapy (CBT), including habit reversal and acceptance and commitment therapy, although there is evidence behind pharmacotherapy, including SSRIs, particularly fluoxetine and sertraline [3-4]. There is also evidence supporting the use of clomipramine, N-acetylcysteine, and olanzapine [4]. The duration of treatment is unknown and based solely on the patient's response to treatment. Trichotillomania can be related to emotions and emotional distress. For many, hair-pulling can be a way of dealing with negative or uncomfortable feelings such as stress, anxiety, tension, boredom, or frustration [4]. Often, there is a heightened sense of tension prior to pulling or when one tries to resist pulling. After pulling their hair, people immediately feel satisfied and have a sense of relief [4]. Thus, they continue to pull their hair to maintain these positive feelings and to manage their negative feelings. The feelings of satisfaction are closely associated with the act of hair pulling, which can result in undesirable aesthetics. The aesthetics of balding in places that cannot be hidden very easily then lead to feelings of shame and low self-esteem.

It is unknown if there is any connection between type 1 diabetes and trichotillomania. Those with diabetes are more likely to suffer from mental disorders [8]. There is a two-fold increase in depression among those with diabetes [8]. Other psychiatric disorders with higher incidences of diabetes include but aren’t limited to obsessive-compulsive disorder, anxiety disorders, schizophrenia, and bipolar disorders. Psychiatric comorbidity in those with diabetes is often associated with higher levels of functional impairment, impaired quality of life, and difficulties with self-care [8-9]. Diabetes can also induce stress, as people often have to be wary of their diet, check blood glucose levels, remember how much insulin to use, and more [9]. This stress can exacerbate underlying mental disorders that can further impair quality of life [9]. This will have unfavorable influences on metabolic glycemic control. In our patient, having insulin-dependent type 1 diabetes was a significant stressor in his life. He also reported past trauma related to a car accident that led to multiple intra-abdominal surgeries. This resulted in feelings of negative self-worth and feeling like a burden to his family. He also had repeated flashbacks to the initial incident that changed the entire course of his life. Thus in order to cope and deal with the negative emotions of stress and to provide a sense of relief, trichotillomania may have developed. However, due to the physical stress from the damage that was caused due to his hair pulling and skin picking, and lack of glycemic control, this could have contributed to him experiencing DKA multiple times via recurrent local site infections. Improvement of therapeutic outcomes is a challenge in the modern healthcare system but can potentially be achieved through a multidisciplinary approach; to treat both his diabetes and mental disorder.

## Conclusions

This case presents a unique exacerbating factor and possible trigger of diabetic ketoacidosis in a type 1 diabetic. Trichotillomania is a mental disorder that is developed as a result of inappropriate coping mechanisms in an individual dealing with stress. Mental disorders are commonly found in those that have diabetes type 1 and type 2. Diabetes is a regimented medical condition that requires strict dietary adherence, routine follow-up, and medication compliance to decrease the risk of adverse acute outcomes such as DKA, hypoglycemia, and even death. It also requires long-term adherence to reduce the overall risks of renal failure, glaucoma, coronary artery disease, gastroparesis, and peripheral neuropathy. There is enough evidence to suggest the psychophysiological relationship between diabetes and mental disorders is markedly important in improving the patient's overall health outcome. As such, it is important to always address psychiatric and medical problems in order to manage a patient’s health properly. Treatment should involve a multidisciplinary approach, including dermatologists, psychiatrists, psychologists, and pediatricians. Further research continues in regards to the pathophysiology, genetic markers, and pharmacotherapy to improve compulsive disorders such as trichotillomania.
